# FN400 amplitudes reveal the differentiation of semantic inferences within natural vs. artificial domains

**DOI:** 10.1038/s41598-018-30684-3

**Published:** 2018-08-17

**Authors:** Changquan Long, Mingming Zhang, Ruifang Cui, Jie Chen

**Affiliations:** 1 Key Laboratory of Cognition and Personality of the Ministry of Education, Southwest University, Chongqing, 400715 China; 2 Key Laboratory for Cognition and Human Behavior of Hunan Province, Hunan Normal University, Changsha, 410081 China

## Abstract

Category-based inferences allow inductions about novel properties based on categorical memberships (*e.g*., knowing all trout have genes [premise] allows us to infer that all fish have genes [conclusion]). Natural (N) and artificial (A) domains are the most obvious and traditional distinctions in categorization. The distinct event-related potential (ERP) responses for N and A domains have not yet been examined during category-based inferences. In this study, the differences between ERP inference parameters within N and A domains were measured during inductive decision processing, while controlling the premise−conclusion similarity and premise typicality between those two domains. Twenty-two adults were asked to make a decision on whether a conclusion was definitely weak, possibly weak, possibly strong, or definitely strong, based on a premise. The behavioral results showed that semantic inferences within the N domain shared similar inductive strength, similar “correct” response rates, and similar reaction times with that within the A domain. However, the ERP results showed that semantic inferences elicited smaller frontal-distributed N400 (FN400) amplitudes within the N domain than within the A domain, which suggested that knowledge of the ontological domain of a category affects category-based inferences, and underlaid the increased categorical coherence and homogeneity in the N as compared to the A categories. Therefore, we have distinguished the cognitive course of semantic inferences between N and A domains.

## Introduction

Categories are essential for human conceptual organization. A central function of categorization is making predictions^1,2^. People can use their categorical knowledge to generalize novel properties from categorical examples, termed category-based inferences. For example, hearing that “All trout [premise category] have novel property X [premise property]”, one may infer that “all fish [conclusion category] would have novel property X [conclusion property]”, because trout belongs to the fish category. This type of inference has various names in the literature, including category-based induction, categorical induction, property induction, feature induction, and induction projection^3^.

One of the most obvious and traditional distinctions in categorization is between the natural (N) and artificial (A) domains. The categories in the N domain include naturally occurring objects (*e.g*., animal), and the categories in the A domain include produced or manufactured objects (*e.g*., tool)^4,5^. Researchers suggest that the N and A domains are distinct in the nature of their ontology^6–11^. The categories in the N domain are generally tightly structured, coherent, and share many similarities (*e.g*., animals have similar internal parts, external structure, and behaviors). In contrast, the categories in the A domain are typically more loosely structured, less homogeneous, and share fewer important features (*e.g*., tools vary in their shape, color, and their constituents).

However, few studies have directly compared the differences between N and A domains during category-based inferences^4,5^. Many previous studies have examined category-based inferences in the N domain; relatively fewer studies include stimuli in both N and A domains during category-based inference tasks. Several studies have shown that knowledge about the nature of a category, including its ontological domain, such as N and A categories, influences inferences^6–8,11^. For example, in a study by Coley *et al*.^8^, only category coherence expectations were found to predict inductive inferences for the N domain, but both knowledge and category coherence expectations predicted inductive inferences for the A domain. Moreover, several studies have found that adults drew more inferences within the N than within the A domain^6–10^. Researchers suggested that the varied patterns of inferences between the N and A domains can be explained by the differences in the categorical coherence and homogeneity between N and A domains (ontological distinction account)^6–8,10,12^.

However, most previous studies have not matched categories between the N and A domain during inferring. For example, some studies have found that the degree of typicality of premise categories affected inferences^13,14^, while few studies have matched the typicality of premise categories between the N and A domains when comparing inferences within these two kinds of categories. Moreover, several theories have suggested that category-based inferences are driven by similarities between premises and conclusions, termed similarity-based models of category-based inferences^15–18^. According to these models, if the degree of similarity between the premise and conclusion under the N and A domain are similar, then the inference decision in both domains would be similar. This hypothesis is supported by Badger and Shapiro^4^. They found that children’s inferences did not vary by domains while controlling for perceptual similarity across those two domains. However, Badger and Shapiro^4^ did not test whether or not adults have similar pattern to children in their study.

The present study therefore aimed to compare adults’ inferences within the N and A domains after matching the degree of premise-conclusion similarity and the degree of premise typicality between them, by using event-related potentials (ERPs). ERPs can uncover cognitive processes in the brain with high temporal sensitivity^19^. Although previous studies have measured the ERP responses to semantic category-based inferences^13,14,20–24^, inferences within the N and the A domains were not directly compared. In the present study, the semantic categories of both the N and A domains were considered. Participants were asked to decide whether a conclusion was definitely weak, possible weak, possible strong, or definitely strong, based on a premise.

Behavioral and ERP responses to the conclusions were recorded and analyzed. As the similarity-based models suggested, we predicted that no significant behavior response differences would be found because the degree of premise typicality and premise-conclusion similarity between the two domains were matched in the present study. However, we predicted that the differences on ERP results would appear, as the ERP measurements are more sensitive to processing differences than behavioral data^19^. More specifically, we predicted that the semantic categories from the N domain would elicit smaller FN400 amplitudes than those from the A domain when making inferences. FN400 is a negative deflection at 200–600 ms, with frontal scalp distribution. Previous ERP studies found that the amplitudes of FN400 were influenced by various factors during sematic category-based inferences^13,20–25^. The attenuated FN400 amplitudes were associated with elevated conceptual priming^26–28^. Inferences within the N domain would therefore elicit smaller N400 amplitudes than those within the A domain because of the enhanced conceptual priming produced by increased categorical coherence and homogeneity in the N compared to the A domain.

## Methods

### Ethical statements

This study was approved by the ethics review board at the Faculty of Psychology, Southwest University, Chongqing, China. Written informed consent was obtained from all participants. The methods were carried out in accordance with the relevant guidelines and regulations.

### Participants

Twenty-two paid undergraduates (mean age: 20.35 years, standard deviation [*SD*]: 1.45; 14 women) were recruited for the ERP study. All participants were right-handed, had normal or corrected-to-normal eyesight, and had no neurological disorders. Prior to the formal ERP experiment, 35 undergraduates (mean age: 20.71, *SD*: 1.81; 17 women) evaluated the degree of experimental items’ familiarity, 35 undergraduates (mean age: 20.57, *SD*: 1.58; 18 women) evaluated the degree of experimental items’ typicality, and another 24 undergraduates (mean age: 20.41, *SD*: 1.32; 15 women) evaluated the degree of similarity between each item and its superordinate category. The evaluators did not participate in the ERP study.

### Experimental design

Two types of premise category domains were used: the N and the A domains. The novel properties were presented by a capital letter and an Arabic number (*e.g*., X1) as blank properties, to reduce the influence of background knowledge^15^. Similar to previous studies^14,20^, a category and a property were presented simultaneously, linked by a blank space to indicate that the category had this novel property (*e.g*., “apple X1” means “apple has novel property X1”.).

Two types of conclusion conditions were also involved: congruent (+) and incongruent (−) conclusions. For congruent conclusions, the conclusion categories included the premise categories (*e.g*., premise: apple X1; conclusion: fruit X1). For incongruent conclusions, the conclusion categories excluded the premise categories (*e.g*., premise: apple X1; conclusion: furniture X1). The premises and conclusions shared the same properties in each trial. Therefore, there were four sub-conditions: congruent conclusions with a natural premise category (N+), congruent conclusions with an artificial premise category (A+), incongruent conclusions with a natural premise category (N−), and incongruent conclusions with an artificial premise category (A−). Similar to previous studies^14,20^, both the conclusion categories and conclusion properties were presented simultaneously, with a question mark (?) to ask participants to judge the inference strength of the conclusions, based on a 4-point Likert scale.

## Materials

All premise categories were basic level categories and were presented with Chinese characters, involving 48 N category items and 48 A category items. The premise categories came from four N (vegetables, birds, fruits, and mammals) and four A (tools, furniture, clothing, and household electrical appliances) categories, and these eight superordinate categories were used as the conclusion categories, and were presented in Chinese characters. Therefore, there were 48 trials for each sub-condition, resulting in a total of 192 trials in the formal experiment. An additional 20 trials (five trials for each sub-condition) were used for training and did not appear in the formal experiment.

Previous studies have shown that the repetitions and frequencies of words affected N400 amplitudes^29–31^. To match the repetition times, for premise categories, every 12 basic level categories came from one superordinate level category. As a result, the N category items involved 12 kinds of vegetables, 12 kinds of birds, 12 kinds of fruits, and 12 kinds of mammals; the A category items involved 12 kinds of tools, 12 kinds of clothing, 12 kinds of furniture, and 12 kinds of appliances. Each premise category appeared twice. For conclusion categories, each superordinate category (vegetables, birds, fruits, mammals; tools, furniture, clothing, and appliances) appeared 24 times: 12 times for congruent condition, and 12 times for incongruent condition. Moreover, we checked the characters’ frequencies (times per 100,000) in the formal experiment against the Corpus Word list (www.cncorpus.org), and found no significant differences between those two domains (N: 51.68 [*SD* = 7.46]; A: 42.09 [*SD* = 6.08]; F [1, 94] < 1, *p* = 0.76).

Before the ERP experiment, the familiarity and typicality of stimuli were evaluated. Participants were required to determine the degree to which they were familiar with the premise category words based on a 5-point Likert scale (1, least familiar; 5, most familiar). For example, participants were instructed to “please judge the degree to which you are familiar with the word ‘apple’ on a 5-point Likert scale; 1 represents least familiar, and 5 represents most familiar”. The familiarity differences between N and A stimuli were not significant (N: 4.20 [*SD* = 0.38], range: 3.10−4.95; A: 4.08 [*SD* = 0.51], range: 3.55 – 4.84; *F* [1, 34] = 1.38, *p* = 0.25). The degree of typicality of the premise categories was evaluated on a 6-point Likert scale (1, least typical; 6, most typical). For example, participants were instructed to “please judge the degree to which you think that apples are typical of fruit on a 5-point Likert scale; 1 represents least typical, and 5 represents most typical”. The typicality for the two types of stimuli were not statistically significantly different (N: 4.80 [*SD* = 0.36], range: 3.53–5.64; A: 4.80 [*SD* = 0.53], range: 4.03–5.48; *F* [1, 34] < 1, *p* = 0.99).

Moreover, the degree of global similarity between each item and its superordinate category was evaluated on a 7-point Likert scale (1, least similar; 7, most similar). For example, participants were instructed to “please judge the degree to which you think that apples are globally similar to fruit on a 7-point scale; 1 represents least similar, and 7 represents most similar”. The global similarities of the two types of stimuli were also not statistically significantly different between items and their superordinate categories, under congruent conclusions (N+ : 5.75 [*SD* = 0.73], range: 4.50–7.00; A+ : 5.83 [*SD* = 0.80], range: 4.71–7.00; *F* [1, 23] = 1.90, *p* = 0.18) as well as under incongruent conclusions (N−: 1.22 [*SD* = 0.33], range: 1.00–2.33; A−: 1.19 [*SD* = 0.36], range: 1.00–2.29; *F* [1, 23] = 1.27, *p* = 0.27).

### Procedures

**Figure 1:**
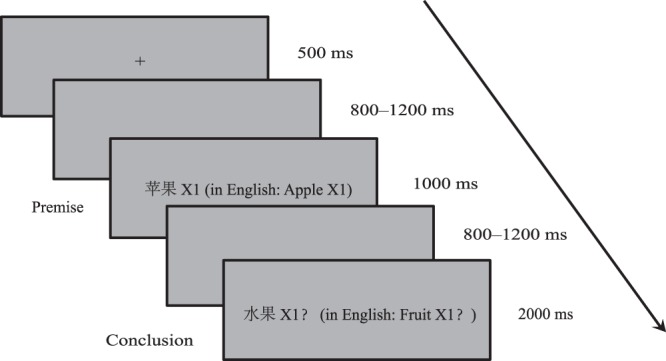
Experimental procedure.

Figure 1 illustrates the experimental procedure. Participants were seated 80 cm in front of a monitor and were instructed to decide on whether a conclusion was definitely weak, possibly weak, possibly strong, or definitely strong, based on a premise. In each trial, a “+” was first presented at the center of the screen. Following the “+”, as shown in Fig. 1, a premise was presented (*e.g*., “apple X1”). After the premise, the conclusions (*e.g*., “fruit X1?”) were presented. Participants were asked to infer the inductive strength of the conclusion based on the premise, by pressing four different number keys representing definitely weak, possibly weak, possibly strong, and definitely strong responses, as accurately and quickly as possible. The experimental trials were divided into four blocks, and were presented randomly. Participants were allowed to rest for 60 s after completing each block. Moreover, to counterbalance key pressing across participants, 11 participants were instructed that 1 was “definitely weak”, 2 was “possibly weak”, 3 was “possibly strong”, and 4 was “definitely strong”, while the other 11 participants were instructed that 1 was “definitely strong”, 2 was “possibly strong”, 3 was “possibly weak”, and 4 was “definitely weak”.

### EEG data acquisition and pre-processing

An electroencephalogram (EEG) was recorded from 64 electrode sites across the scalp using a Neuroscan cap (Neuroscan, Herndon, VA, USA) with Ag/AgCl electrodes while participants responded to the conclusions. The electrodes were positioned according to the International 10–20 system. A ground electrode was placed at the middle of FPz and Fz. The recording was referenced to an electrode between Cz and CPz. A vertical electrooculogram (EOG) was recorded supra- and infra-orbitally at the left eye; and the horizontal EOG was recorded from the left versus the right orbital rim. The EEG and EOG were amplified using a SynAmps2 amplifier (Neuroscan) and digitized in a 500-Hz sample size. The EEG and EOG were amplified with a band-pass filter from 0.05 to 200 Hz in AC mode. All interelectrode impedances were maintained below 5 kΩ. Off-line analyses were performed in MATLAB (MathWorks, Natick, MA, USA) using the EEGLAB^32^ and ERPLAB toolboxes^33^. EEG data were filtered using IIR-Butterworth filters with half-power cutoffs at 0.1 and 30 Hz (roll-off = 12 dB/oct)^19^. Independent component analysis (ICA) was subsequently performed to correct components associated with eye movements and eye-blinks. Then, the ICA-corrected EEG data were re-referenced to the average of the left and right mastoids^19^.

The epochs were segmented time-locked to the conclusions. The “definitely strong” and “possibly strong” responses to congruent conclusions, and the “definitely weak” responses and “possibly weak” responses to incongruent conclusions were identified as “correct” responses. Only “correct” responses were overlapped and averaged. Each epoch was 1200 ms, including a 200-ms pre-stimulus baseline correction. Trials were excluded as noise by using peak-to-peak amplitude sliding window method^19^, with a window-width of 200 ms, a window step of 100 ms, and a threshold of 65 μV. This resulted in 36.82 trials (*SD*: 5.03) for N+, 38.27 trials (*SD*: 5.17) for N−, 37.45 trials (*SD*: 4.65) for A+, and 37.68 trials (*SD*: 5.75) for A− conditions. There was no significant difference among the number of trials under four conditions.

### Data analyses

Behavioral and ERP responses to the conclusions were analyzed. Two-factor repeated-measures analyses of variances (ANOVAs) were used to analyze the response strength, “correct” response rates, and reaction times of “correct” responses. The factors were domains (N or A) and congruencies (+ or −). For the response strength, the “definitely weak” response was defined as a score of 1, the “possibly weak” response was defined as a score of 2, the “possibly strong” response was defined as a score of 3, and the “definitely strong” response was defined as a score of 4.

**Figure 2:**
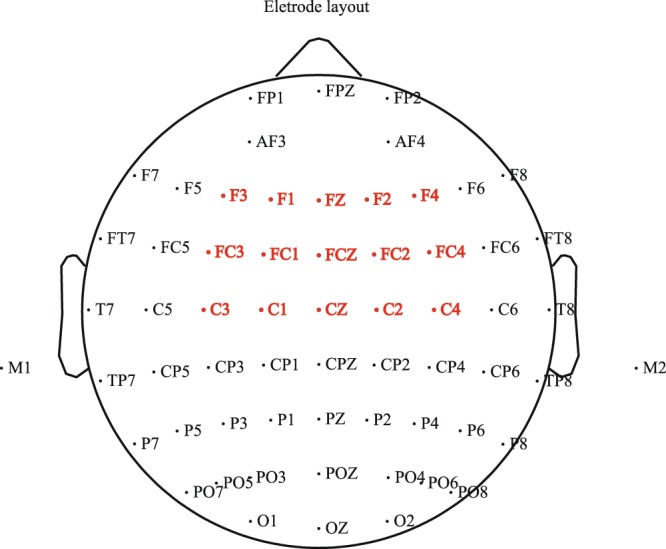
The electrode layout and the region of interest (ROI). The red cluster is ROI that be used for the further two-factor repeated-measures analyses of variances (ANOVAs) for ERP data.

Figure 2 shows the electrode layout and the region of interest (ROI). According to previous studies^14,20,28,34^ and visual observation, the F3, F1, Fz, F2, F4, FC3, FC1, FCz, FC2, FC4, C3, C1, Cz, C2, and C4 electrode sites at the fronto-central region were selected, and the mean amplitudes of FN400 were measured in a 250–450-ms time-window after conclusion onset. To illustrate relatively precise temporal courses, data analysis was executed for each 50 ms. Moreover, to increase the statistical strength, and to reduce false effects^35^, these electrodes were collapsed by averaging. The 2-factor repeated-measures ANOVAs were used to analyze the mean amplitudes of FN400. The factors were domains (N or A) and congruencies (+ or −). For all analyses, the *p*-values of *F* tests were corrected for deviations using the Greenhouse–Geisser method, and *post-hoc* multiple comparisons were performed by the Bonferroni procedure. For the analysis of the mean amplitudes of FN400 at 250–450-ms, the *p*-values were corrected using the false discovery rate for multiple comparisons (*p* < 0.05 determined statistical significance).

## Results

### Behavioral results

**Figure 3:**
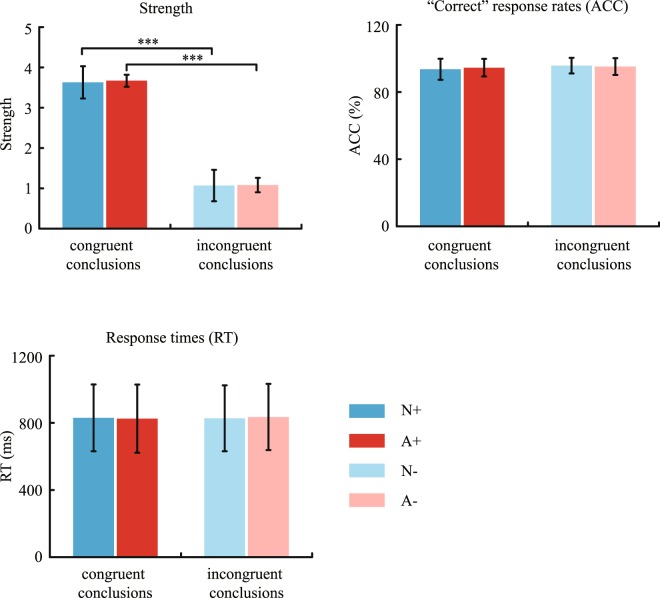
The averaged inference strength, “correct” response rates (ACC), and reaction times (RTs) of “correct” responses for each sub-condition. N+ indicates “congruent conclusions with a natural premise category”; A+ indicates “congruent conclusions with an artificial premise category”; N− indicates “incongruent conclusions with a natural premise category”; A− indicates “incongruent conclusions with an artificial premise category”. Error bars represent mean ± s.e.m. ***p < 0.001.

Figure 3 shows the averaged inference strength, “correct” response rates (ACC), and reaction times (RTs) of “correct” responses for each sub-condition. For averaged inference strength, no significant main effect of domains (*F* [1, 21] = 3.19, *p* = 0.09, η^2^_p_ = 0.13) suggests that N arguments had similar response scores to A arguments. A significant main effect of congruencies (*F* [1, 21] = 675.76, *p* < 0.001, η^2^_p_ = 0.97) suggests that the response scores under congruent conclusions were greater than those under incongruent conclusions. The interaction of domains and congruencies did not reach significance (*F* [1, 21] < 1, *p* = 0.45, η^2^_p_ = 0.03).

For both “correct” response rates and reaction times of “correct” responses, the main effects of domains (ACC: *F* [1, 21] < 1, *p* = 0.85, η^2^_p_ < 0.01; RT: *F* [1, 21] < 1, *p* = 0.88, η^2^_p_ < 0.01) and congruencies (ACC: *F* [1, 21] = 2.39, *p* = 0.14, η^2^_p_ = 0.10; RT: *F* [1, 21] < 1, *p* = 0.87, η^2^_p_ < 0.01) were not significant. The interactions between domains and congruencies also did not reach significance (ACC: *F* [1, 21] < 1, *p* = 0.45, η^2^_p_ = 0.03; RT: *F* [1, 21] < 1, *p* = 0.41, η^2^_p_ = 0.03). Altogether, inferences within the N and A domain categories had similar response strengths (N+: *M* = 3.63, *SD* = 0.40; A+: *M* = 3.67, *SD* = 0.39; N−: *M* = 1.07, *SD* = 0.15; A−: *M* = 1.08, *SD* = 0.18), “correct” response rates (N+: *M* = 93.42%, *SD* = 6.15%; A+: *M* = 94.32%, *SD* = 5.05%; N−: *M* = 95.50%, *SD* = 4.45%; A-: *M* = 94.89%, *SD* = 4.72), and reaction times for correct responses (N+: *M* = 830 ms, *SD* = 199 ms; A+: *M* = 825 ms, SD = 196; N−: *M* = 827 ms, *SD* = 203 ms; A−: *M* = 835 ms, *SD* = 197 ms).

### ERP results

**Figure 4:**
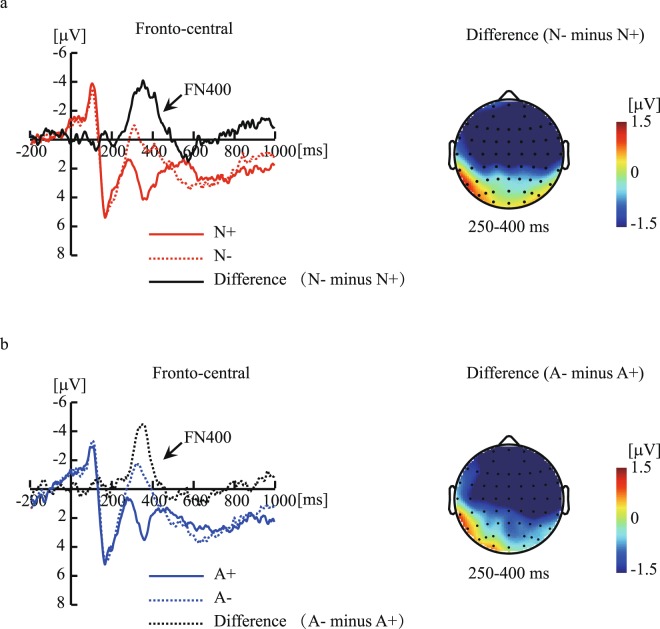
The ERP responses to the congruency effects. Figure 4a shows the grandaveraged waveforms elicited by N+ and N− arguments and the difference waveform (N− minus N+) in the fronto-central region, and the topography of the difference waveform at 250–400-ms. Figure 4b illustrates the grand-averaged waveforms elicited by A+ and A− arguments and the difference waveform (A− minus A+) in the fronto-central region, and the topography of the difference waveform at 250–400-ms. N+ indicates “congruent conclusions with a natural premise category”; A+ indicates “congruent conclusions with an artificial premise category”; N− indicates “incongruent conclusions with a natural premise category”; A− indicates “incongruent conclusions with an artificial premise category”.

**Figure 5:**
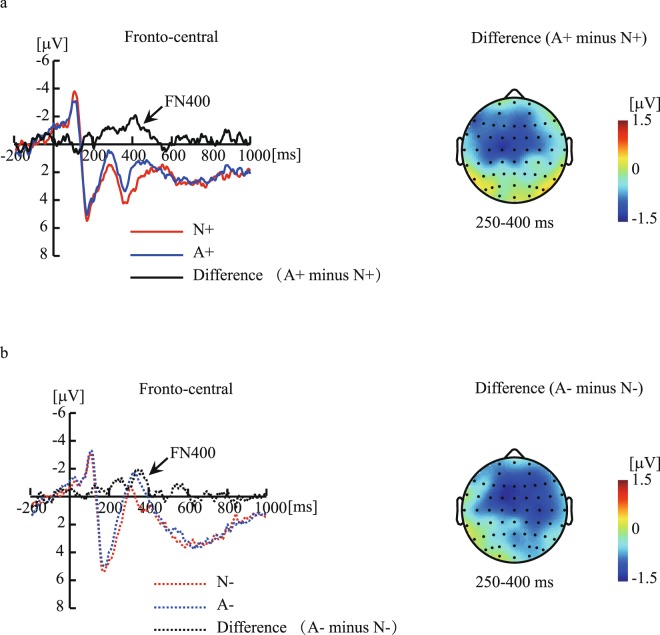
The ERP responses to the domain effects. Figure 5a shows the grandaveraged waveforms elicited by N+ and A+ arguments and the difference waveform (A+ minus N+) in the fronto-central region, and the topography of the difference waveform at 250–400-ms. Figure 5b illustrates the grand-averaged waveforms elicited by N− and A− arguments and the difference waveform (A− minus N−) in the fronto-central region, and the topography of the difference waveform at 250–400-ms. N+ indicates “congruent conclusions with a natural premise category”; A+ indicates “congruent conclusions with an artificial premise category”; N− indicates “incongruent conclusions with a natural premise category”; A− indicates “incongruent conclusions with an artificial premise category”.

Figure 4 shows the ERP responses to the congruency effects. Figure 5 shows the ERP responses to the domain effects. Table 1 shows the results of two-way repeated-measures ANOVAs during 250–450-ms for every 50-ms.

**Table 1:** Results of 2-way repeated-measures ANOVAs of the 250–450-ms time window for each 50-ms.

Parameter		Time window (ms)			
		250–300	300–350	350–400	400–450
Domains (D)	*F*	7.58	8.24	11.65	2.74
	*p*	0.01	0.01	0.01	0.11
	*Corrected p*	**0.02**	**0.02**	**0.01**	0.17
	η^2^_p_	0.27	0.28	0.36	0.12
Congruencies (C)	*F*	8.30	41.14	23.23	3.28
	*p*	0.01	<0.001	<0.001	0.08
	*Corrected p*	**0.02**	**<0.001**	**<0.001**	0.14
	η^2^_p_	0.28	0.66	0.53	0.14
D*C	*F*	0.01	0.28	0.006	1.49
	*p*	0.91	0.60	0.94	0.24
	*Corrected p*	0.94	0.72	0.94	0.32
	η^2^_p_	<0.01	0.01	<0.01	0.07

The **bold** font illustrates a significant *corrected p* value.

During the 250–300-ms, 300–350-ms, and 350–400-ms time window, the interaction between domains and congruencies was not significance (Table 1). However, a main effect of domains reached significance, revealing that A arguments elicited more negative amplitudes than N arguments. Also, a main effect of conclusion congruency reached significance, revealing that incongruence arguments elicited more negative amplitudes than congruency arguments (Table 1). During the 400–450-ms time window, no significant conclusion congruency effects and domain effects were found (Table 1). Altogether, the results indicated that A arguments elicited more negative amplitudes than N arguments under both congruent and incongruent conditions, and incongruent conclusions elicited more negative amplitudes than congruent conclusions under both N and A arguments in the 250–400-ms time window at the fronto-central region.

## Discussion

The present study distinguished between the ERP responses to semantic inferences in the N and A domains after matching the degree of premise-conclusion similarity and the degree of premise typicality between the two domains. There were no significant differences in inference strength, “correct” response rates, and reaction times of “correct” responses between inferences in the N and A domains. However, the ERP results, in terms of the FN400 amplitudes, differentiated the inferences between the N and A domains.

In line with the prediction of the similarity-based models of semantic inference^15–17^, the behavioral results of this study showed that semantic inferences within the N and A domains shared similar inference strength, “correct” response rates, and reaction times of “correct” response. The similarity-based models of semantic inference^15–17^ suggested that premise–conclusion similarity and premise typicality play central roles during category-based inferences. Badger and Shapiro^4^ found that 4–9-year-old children did not vary their inferences between the N and A domains while matching over perceptual similarity across those two domains. Extending to Badger and Shapiro^4^, in the present study, adults have no significant behavior differences between inferences in the N and A domains, after controlling the degree of premise–conclusion similarity and the degree of premise typicality between those two domains.

However, a difference in semantic inferences in the N and A domains was found in the 250–400-ms time window, suggesting that ERP measurements are more sensitive to processing differences than behavioral data^19^. In the present study, incongruent conclusions elicited greater frontal-distributed negative deflections than did congruent conclusions in the 250–400-ms time window. The current ERP effects on conclusion congruent effects were similar to previous FN400 effects related to conclusion congruency effects during category-based induction^20,21^. Moreover, in the present study, ERP responses to domain effects were found, and A arguments elicited greater negative deflections than did N arguments. The ERP responses to the domain effects differed slightly from the FN400 effects shown by the conclusion congruent effects, as the effect sizes were smaller for the former than for the later in the 300–400-ms time window. However, the ERP responses to the domain effects strongly overlapped the FN400 effects in terms of time windows and distributions. From this observation, we inferred that the ERP responses to both conclusion congruence effects and domain effects are the FN400 effects.

The current FN400 effects are not due to differences in repetition or word frequency, because the number of repetitions and word frequency were controlled for both domains in the present study. Rather, we argue that the current FN400 effects between the N and A domains might be related to the ontological distinction between the N and A domains. FN400 is related to conceptual priming, with smaller N400 amplitudes in increased conceptual priming^26–28^. According to the ontological distinction account, the categories in the N domain are more coherent and homogenous than those in the A domain^7–10^, which may increase conceptual priming for categories from the N domain rather than those from the A domain. As a result, the semantic inferences in the N domain elicited smaller FN400 amplitudes than did those within the A domain.

Another potential explanation for the different FN400 effects on semantic inference in the N and A domain is that the FN400 amplitude was associated with the degree of similarity. Previous studies have suggested that the FN400 amplitudes are associated with familiarity, with greater familiarity eliciting smaller FN400 amplitudes^34,36–39^. Familiarity can reflect an assessment of the global similarity between items^40,41^. However, in present study, the degree of premise–conclusion similarity within the N domain was similar to that within the A domain. Thus, the observed FN400 effects demonstrated here were not related to differences in the degree of premise–conclusion similarity between the two domains.

The distinctive patterns between behavior and ERP results for inferences within N vs. A domains do not suggest that the similarity-based account and the ontological distinction account are mutually exclusive. Knowledge can form on the basis of perceptual and conceptual experience^18^. The different representations and processing of ontological knowledge for N and A domains can be shaped by their perceived perceptual and conceptual similarity during learning. The ontological distinction was shown by FN400 amplitudes when the premise typicality and premise–conclusion similarity were controlled, providing further evidence that the ontological distinction is a parameter influencing category-based inferences.

The effects of the FN400 amplitudes between the N and A domains during inference-making was inconsistent with previous studies, and provided further evidence that N400 effects between the N and A domains were task-dependent. Previous studies have compared the ERP responses between N and A domains, but found inconsistent results in terms of N400 amplitudes. Several studies have reported that the N domain elicited greater N400 amplitudes in the frontal region, and elicited smaller N400 amplitudes in the central and parietal regions, as compared to A domain^42–44^. However, Satori *et al*.^45^ and Fuggetta *et al*.^46^ did not find significant differences in N400 between N and A domains. In the present study, the N400 difference pattern did not correspond with these previous studies.

Devlin *et al*.^47^ pointed out that the inconsistency within the differences between N and A domains was due to task differences. In previous studies, various tasks were used, involving semantic categorization^42,44,48–51^, lexical decision^43^, picture-word matching^46^, semantic priming^51,52^, feature-category matching^45^, picture naming^49^, gender decision^44^, and visual/functional judgment^48^. In these studies, participants were required to assess what they had learned. In the present study, a categorical inductive inference task was used in which participants were required to go beyond what they had learned^3,53^. During categorical inductive inferences, more flexible representations were produced, involving similarity, categorical inclusion, and causal-explanatory categorical relationships to determine inferences^54–56^. Distinct task demands may therefore explain inconsistency of the results in the literature.

However, the ERP parameter differences between inferences for N and A categories did not provide strong evidence for the conceptual representation hypotheses. Whether semantic memory representations of N and A categories are supported by single or multiple cortical systems is debated^57–59^. The single cortical system hypothesis^60–63^ suggests that various activation patterns correspond to different semantic properties in the perceptual and motor systems of the brain. However, the objects’ sensory or motor features will be transformed into a common amodal representation. The multiple cortical systems hypothesis^57^ proposes that semantic memories are organized in modality-specific semantic subsystems. The multiple cortical systems hypothesis involves two distinct theoretical approaches. The whole-object account suggests that each semantic category has its own discrete neuroanatomical structure in the brain^64^, and the feature-based account suggests that sematic categories are grounded in different object feature types (*e.g*., visual, motor)^58^.

Ković *et al*.^63^ suggested that ERP parameter differences between N and A categories may not reflect modality specificity, but variability or homogeneity in those two categories, as suggested by the present study. However, other studies have suggested that N400 differences between N and A categories support modality-specific hypotheses^42–44,48^. By this logic, the significant FN400 amplitude differences between N and A categories in our study provide further evidence for modality-specific hypotheses of conceptual representations. Therefore, a limitation of our study is that it did not provide strong evidence for either amodal or modality-specific conceptual representation hypotheses. More research is needed on this issue.

## Conclusions

The present study contributed to the elucidation of the complex differential processes underlying semantic inferences within N and A domains. The behavioral results showed that semantic inferences within N and A domains shared similar inference strength “correct” response rates, and reaction times after matching the degree of premise–conclusion similarity and the degree of premise typicality between the two domains. However, the ERP results showed that differences existed during semantic inferences within the N and A domain. The N domain elicited smaller FN400 amplitudes than the A domain, suggesting a role for increased categorical coherence and homogeneity in the N domain, as compared to the A domain, during semantic inferences.
